# A new journal – "Theoretical Biology and Medical Modelling"

**DOI:** 10.1186/1742-4682-2-21

**Published:** 2005-06-12

**Authors:** Denys N Wheatley

**Affiliations:** 1 BioMedES, Leggat House, Keithhall, Inverurie, Aberdeen AB51 0LX, UK

## Abstract

Biology has a conceptual basis that allows one to build models and theorize across many life sciences, including medicine and medically-related disciplines. A dearth of good venues for publication has been perceived during a period when bioinformatics, systems analysis and biomathematics are burgeoning.

Steps have been taken to provide the sort of journal with a quick turnaround time for manuscripts which is online and freely accessible to all readers, whatever their persuasion or discipline. We have now been running for some time a journal which has had many good papers presented pre-launch, and a steady stream of papers thereafter. The value of this journal as a new venue has already been vindicated.

Within a short space of time, we have founded a state-of-the-art electronic journal freely accessible to all in a much sort-after interdisciplinary field that will be of benefit to the thinking life scientist, which must include medically qualified doctors as well as scientists who prefer to build their new hypotheses on basic principles and sound concepts underpinning biology. At the same time, these principles are not sacrosanct and require critical analysis. The journal  promises to deliver many exciting ideas in the future.

## Introduction

Several stories have been told about theorists; here are two.

Two proponents of ideas from opposing schools of thought took advice from a third party. To A he said she ought to listen to the arguments propounded by B and not just dismiss them out of hand; to B he said he should listen to the arguments of A and not simply dismiss them. To them both, he said "I have been fair with you both, since in all probability you are both wrong!"

The other story shows up the facileness of some arguments (or their proponents): One day, Socrates was discussing Sod's law, also well known in the world of the Ancient Greeks, who like us all buttered their bread on one side. The law – also know as Murphy's law – states that if dropped, the piece invariably lands butter-side down. Next day one of his disciples came rushing to him, remarking that at home in the evening he had buttered a piece of bread, dropped it, and to his amazement it landed butter-side up! Socrates thought for a moment before severely rebuking him "you fool; you buttered the wrong side!"

How this new journal came about is an interesting story. Continued dissatisfaction with other theoretical journals, especially their slowness, and problems at *Comments in Theoretical Biology*, were largely the precipitating factors. I also had colleagues at the Torino Politechnico supporting me in the need for a vehicle to publish cancer modelling problems as part of developing a European network of excellence. This was reinforced by an editorial in *Nature *[1] starkly pointing out the lack of a reasonable focus for informatics on cancer and the need for a modeling forum, at least in Europe, to help oncologists, but equally many other scientists and doctors. Dr David Eagleman [2] made a similar highly relevant point about theoretical science. I suggested that the network of excellence we were striving for in our European Consortium ought to have a web presence, so why not have an online journal? That is why I started one. Having undertaken primarily the role of founding editor, my job was to get the journal up and running, and provide the editorial facilities. We have the assured services of Dr Agutter and Ms Angela Panther as managing editor and editorial officer, respectively, overseeing the smooth running of the journal on a daily basis.

Paul and I have published many papers in the field of theoretical biology and medicine, which are disseminated over a wide range of journals that do not allow interested parties to keep many of them easily in focus. Our experience in producing scientific papers as well as writing them comes from a knowledge of e-publications, having one of the first specialist (for which now read "independent") journals to be published by BioMed Central (BMC)^3 ^online, *viz. Cancer Cell International *****

## The nature of the journal

Speculation within limits, theorizing, and philosophizing are part and parcel of biology and medicine, which are in turn part of life. Having established certain facts or observations, we can employ deductive or inductive reasoning depending on the number of such pieces of information – as Bacon suggested – to reach certain tenuous conclusions. From this position, we create hypotheses, the very best being those that can be tested, not the unapproachable ones that will remain for ever. Biology has a broad coverage, while medicine in contrast has a much narrower focus, because its concepts must be the very same ones as biology in general is built. These in turn have many of their groundings in chemical and particularly physical laws, but not all. The law of mass action cannot be applied under the conditions of synthesis of a new molecule of DNA. In addition to theorizing in medicine, we can also build models that have clinical relevance to help us understand such problems as the spread of diseases throughout the body.

The opening up of bioinformatics, the proliferation of databases on genes, proteins, metabolites and so on, have created a climate in which many scientists can puddle to their heart's content to make significant and meaningful correlations. New ideas spring from some of these, just as old ideas can find evidence suddenly becoming available that was needed years ago to support them. Interdisciplinary work is now highly fashionable; and mathematical biology needs to become a force *within *biology, not a peripheral, elitist activity. And so on; I rest my case.

The new journal on the web will consider high quality, peer-reviewed theoretical papers. It will also seek to provide new ideas that may be quite off the main-stream of biomedicine; after all, today's "crazy" notion has a not so infrequently had the habit of becoming tomorrow's received wisdom. My two colleagues, who are also Editors-in-Chief are Pier Paolo Delsanto in Turin and Hans-Peter Meinzer in Heidelberg. The editorial board includes many people well known to those who already publish in theoretical journals.

The advantages of using the web, if not already self-evident, will become clear to you all once you appreciate what a wonderfully interactive tool it affords. Debates on papers can be posted along with referees' criticisms, if they so choose. Commentaries can be put up online, much as already done in BioMed Central's *Journal of Biology. *But we also want theoretical work that is topical. Often editors see no need to move theoretical papers quickly through the publication process. Indeed, the only ones that get held up even longer are papers dealing with the history of biology, many of which often take years to complete the acceptance and publication processes, my last one taking very close to two years.

Many issues arise on which we are forced through incomplete knowledge to hypothesize about causes, such as the spread of SARS. When SARS arose, ideas needed to be discussed as critically as possible, and an online journal has almost a similar capacity to handle "copy" in the way that newspaper handles it, although there is a world of difference between the editorial role of "*Tbiomed*" (our shortened title and URL name) and one in a tabloid newspaper that is there to feed on drama, rumour and innuendo to sell their products rather than on sound facts and rational arguments. No one is selling anything in science, especially that which gets published and is accessible to everyone free of charge online. So a theoretical journal that is state- of-the-art emanating from BioMed Central offers exactly what the doctor ordered, PhD or MD. I hope you will make maximum use of it, and send papers that will stir the thoughts of others.

We accept that there is the old thorny problem of impact factors. My feeling is that no one scores heavily regarding grant proposals from publishing a paper with your latest and greatest hypothesis. The grant-awarding agencies provide funds for *testing *hypotheses, not formulating them. Therefore in most cases publishing in *Tbiomed *is unlikely to affect your street-credibility when it comes to grant proposals; indeed it can help because the hypothesis you want to test is out there online for all to read and explore. The other issue is that impact factor continues to relate to journal performance and not directly to the quality of the *individual *article. Because all new papers will soon be completely accessible via the web, the paper itself can be rated rather than the journal that holds it. An example of this is Faculty of 1000, published by BioMed Central, where experts can pick up and extol the virtues of an individual paper, no matter in which journal it was published.

## A call for papers

Our policy will be liberal, but we will not publish substandard arguments, diatribes and purely rhetorical pieces. Peer review will be strict, but we also will give space to people with unconventional theories that have been presented with sound logical reasoning. So here is a call for papers:

So often we hear people say "...well, in theory, you may be right!"

– so why not submit your hypotheses to one of BMC's newest journals and find out what others think of them.

## Advantages of publishing in Tbiomed

• **Interactiveness**

• **Accessible to all**

• **No charges to authors from all BioMed Central subscribing member institutions**

• **Presentation of complex equations and figures simple and accurate**

• **Peer review criticisms can be published alongside articles**

• **Running commentaries can be made on published papers**

• **No restriction on length, although brevity of expression will be sought**

• **Colour can be used as extensively as required**

• **Is a journal that forms a focus and interface for bioinformatics and multidisciplinary articles**

• **Topical matters requiring theoretical consideration will be published quickly**

• **Juxtaposition of biology and medicine is intentional to spawn more and better interactiveness**

• **Articles can be accessed and disseminated freely, meaning no reprint costs**
